# Prepandemic Risk Factors for Disabling Long COVID: A Prospective Cohort Analysis

**DOI:** 10.1155/jotm/9396282

**Published:** 2026-04-10

**Authors:** Yusuff Adebayo Adebisi

**Affiliations:** ^1^ College of Social Sciences, University of Glasgow, Glasgow, UK, gla.ac.uk; ^2^ Scottish Centre for Administrative Data Research, University of Glasgow, Glasgow, UK, gla.ac.uk

**Keywords:** long COVID, prospective cohort, public health, risk factors

## Abstract

**Introduction:**

Disabling long COVID, characterised by persistent symptoms that limit daily functioning, has emerged as an important public health concern. However, prospective evidence on predisposing risk factors remains limited.

**Methods:**

This study used prospective data from the UK Household Longitudinal Study, linking prepandemic baseline information collected in Wave 10 (2018–19) with follow‐up data from Wave 14 (2022–23). The analytic sample comprised 12,033 adults aged ≥ 16 years who participated in both waves and self‐reported a positive COVID‐19 test at follow‐up. The primary outcome, disabling long COVID, was defined as symptoms lasting more than 12 weeks that impaired day‐to‐day activities. Prepandemic sociodemographic, health and psychosocial factors assessed at baseline were included as predictors. Associations were estimated using modified Poisson regression with robust standard errors to calculate adjusted relative risks (RRs).

**Results:**

Disabling long COVID was reported by 690 individuals (5.7%). Higher risk was observed among women (RR 1.26; 95% CI 1.08–1.48) and adults aged 30–49 (RR 1.38; 95% CI 1.10–1.73) or 50–69 (RR 1.28; 95% CI 1.01–1.62) years, compared with those aged 16–29 years. Additional risk factors included pre‐existing health conditions (RR 1.31; 95% CI 1.10–1.56), poor self‐rated health (RR 1.78; 95% CI 1.40–2.25), psychological distress (RR 1.44; 95% CI 1.21–1.72) and poorer sleep quality (fairly bad: RR 1.92; 95% CI 1.45–2.56; very bad: RR 1.96; 95% CI 1.37–2.81), compared with very good sleep quality. Compared with non‐White participants, White participants had lower risk (RR 0.75; 95% CI 0.61–0.92), while moderate (RR 0.76; 95% CI 0.62–0.93) and high (RR 0.81; 95% CI 0.67–0.98) income satisfaction, compared with low‐income satisfaction, were protective. Stratified analyses showed that the effects of rural residence (*p* for interaction = 0.011) and income satisfaction (*p* = 0.009) differed significantly by sex, with weaker evidence for age (*p* = 0.095) and self‐rated health (*p* = 0.061).

**Conclusion:**

Prepandemic health, socioeconomic and psychological vulnerabilities were independently associated with disabling long COVID, with distinct sex‐specific patterns of risk.

## 1. Introduction

Since the onset of the COVID‐19 pandemic, the global health community has increasingly turned its attention to the long‐term consequences of SARS‐CoV‐2 infection beyond the acute phase of illness [1]. One of the most pressing concerns is the emergence of long COVID, also known as post‐COVID‐19 condition, which is now recognised as a constellation of symptoms persisting for more than 12 weeks after initial infection in the absence of an alternative diagnosis [1]. These symptoms range from fatigue, breathlessness and cognitive impairment to musculoskeletal pain, gastrointestinal issues and psychological distress [1, 2]. Although the precise mechanisms remain unclear, long COVID is believed to result from a combination of viral persistence, immune dysregulation and autonomic dysfunction [1].

While many individuals report mild or gradually improving symptoms, a substantial subset experience debilitating impairments [3, 4]. Recent data estimate that 3.3% of people living in private households in England and Scotland, approximately two million individuals, self‐report long COVID. In nearly three‐quarters of these cases, day‐to‐day activities are adversely affected, with roughly one in five reporting that their activities are ‘limited a lot’ [2]. This condition, here referred to as disabling long COVID, is increasingly regarded as a significant public health concern, with implications for workforce participation, health utilisation and long‐term disability support systems [5, 6]. Despite growing policy interest, systematic evidence on who is most at risk of developing disabling long COVID remains limited.

Much of the current understanding of long COVID stems from clinical cohorts, electronic health records or convenience samples, which, while valuable, are often restricted to individuals who seek care and may underrepresent marginalised groups or those with less access to health care [1]. Moreover, many studies fail to distinguish between the presence of persistent symptoms and their functional impact—a distinction critical for understanding health inequalities and guiding resource allocation [7, 8]. Long COVID symptoms, such as fatigue and cognitive dysfunction, can be widespread, but their capacity to limit activities of daily living varies considerably [9]. Consequently, the UK Office for National Statistics (ONS) and NHS England have begun to differentiate between ‘non‐disabling’ and ‘disabling’ forms of long COVID, the latter being characterised by a self‐reported reduction in daily functioning [10]. This functional framing aligns with the broader conceptualisation of disability within global health and human rights frameworks, including the International Classification of Functioning, Disability and Health (ICF) [11]. Yet, few studies have investigated disabling long COVID using prospective data that links prepandemic baseline information with postinfection outcomes.

This study aims to identify prepandemic risk factors associated with disabling long COVID in a large population‐based cohort of UK adults. Using longitudinal data, this study estimates the association between factors measured before the pandemic and the development of disabling long COVID at follow‐up. It further examines whether these associations differ by sex.

## 2. Methods

### 2.1. Study Design, Data Source and Study Population

This study is a prospective cohort analysis using data from the UK Household Longitudinal Study (UKHLS), also known as Understanding Society. UKHLS is a nationally representative panel survey that follows individuals in households across the United Kingdom [12]. It collects annual data on a wide range of topics, including health, education, employment and behaviours [12]. The survey uses a complex stratified, clustered sampling design and includes ethnic minority boost samples to ensure broad representativeness [12].

Wave 10 (2018–19) served as the prepandemic baseline and Wave 14 (2022–23) as the postpandemic follow‐up [13]. Because UKHLS is an open cohort, ‘refreshment’ samples are periodically recruited to offset attrition and maintain representativeness. Accordingly, Wave 14 contained slightly more adult respondents (35,471 aged ≥ 16 years) than Wave 10 (34,319) [13]. All respondents were linked across waves via a unique personal identifier. Of the 46,405 unique individuals observed in either wave, 23,385 participated in both waves and formed the longitudinal sample for this analysis.

Among the longitudinal sample, 12,036 participants self‐reported a positive COVID‐19 test and provided responses to the long COVID questions in Wave 14. The analysis included individuals who (i) participated in both Wave 10 (2018–19) and Wave 14 (2022–23), (ii) reported a positive COVID‐19 test and (iii) provided valid responses to both long COVID‐related questions. Three individuals were excluded because they were interviewed by proxy or answered ‘don’t know’ or ‘refused’ when asked whether their symptoms limited their ability to carry out daily activities. After these exclusions, the final analytic sample consisted of 12,033 adults.

### 2.2. Outcome Variable

The primary outcome was disabling long COVID, assessed at follow‐up in Wave 14 (2022–23). Respondents who reported a previous COVID‐19 infection were asked: ‘Did you have coronavirus symptoms that lasted more than 12 weeks (after the initial infection)?’ Those who responded ‘Yes’ were further asked whether their symptoms reduced their ability to carry out day‐to‐day activities. Disabling long COVID was defined as a binary variable coded 1 for individuals who reported both (i) having long COVID and (ii) that their symptoms limited daily activities. All other participants, including those who had COVID but did not develop long COVID, or who had nondisabling long COVID, were coded 0. Participants who responded via proxy or selected ‘don’t know’ or ‘refused’ were excluded. This operational definition reflects public health standards and clinical definitions of functionally limiting post‐COVID‐19 conditions used by the UK ONS and NHS England [2, 10].

Guided by the ICF and the Disablement Process Model [11], disabling long COVID is conceptualised not merely as persistent symptoms but as a condition that impairs a person’s capacity to function in daily life. The ICF framework defines disability as a limitation in activity or participation arising from a health condition, shaped by personal, social and environmental contexts. The Disablement Process Model further describes disability as the cumulative outcome of individual‐level risks (e.g. age, health and psychological distress) and structural factors (e.g. socioeconomic disadvantage or geographic context) that either buffer or accelerate the transition from illness to functional impairment. These frameworks informed both the outcome definition and the choice of prepandemic predictors.

### 2.3. Predictor Variables

A range of prepandemic predictors were included based on theoretical relevance, prior evidence and data availability in Wave 10 of the UKHLS. In line with the ICF and the Disablement Process Model [11], these variables were grouped into three conceptual domains: predisposing characteristics (age, sex and ethnicity); baseline health status (long‐standing illness, self‐rated health and smoking status); and psychosocial or contextual factors (income satisfaction, psychological distress, sleep quality and area of residence). These categories reflect the view that disabling long COVID arises from the interplay between biological vulnerability, prior health and social context, not from infection alone.

Age was grouped into four categories: 16–29, 30–49, 50–69 and 70 years or older. Sex was coded as male or female. Ethnicity was classified as White (including British, Irish and other White backgrounds) versus non‐White (including individuals identifying as mixed, Asian, Black, Arab or other ethnic groups). Area of residence was classified as urban or rural, based on derived household location data. Income satisfaction, originally measured on a 7‐point scale ranging from ‘completely dissatisfied’ to ‘completely satisfied’, was recoded into three categories: low (completely, mostly or somewhat dissatisfied); moderate (neither satisfied nor dissatisfied, or somewhat satisfied); and high (mostly or completely satisfied).

Long‐standing illness (pre‐existing health condition) was a binary indicator of whether participants reported any long‐term physical or mental health condition or disability. Self‐rated general health was recoded as excellent or very good, good, or fair or poor. Psychological distress was assessed using the General Health Questionnaire (GHQ‐12), with scores ≥ 4 classified as GHQ caseness (indicating likely clinical‐level distress) and < 4 as no distress. Current smoking status was categorised as Yes or No. Sleep quality was assessed at baseline using a self‐reported measure categorised as ‘Very good’, ‘Fairly good’, ‘Fairly bad’ or ‘Very bad’, based on responses to the question about overall sleep quality.

Where applicable, missing responses for predictor variables were retained and included in the model as distinct categories.

### 2.4. Statistical Analysis

Descriptive statistics were used to compare baseline characteristics between individuals with and without disabling long COVID. Frequencies and percentages were reported for categorical variables, and chi‐square tests were used to assess group differences. The distribution of specific long COVID symptoms among participants reporting disabling symptoms was also summarised.

To estimate associations between baseline characteristics and disabling long COVID, Poisson regression models with robust standard errors were fitted to calculate relative risks (RR) and 95% confidence intervals. Multivariable models included age group, sex, ethnicity, area of residence, income satisfaction, smoking status, long‐standing illness, self‐rated general health, psychological distress and sleep quality. Because the aim was descriptive epidemiology, characterising patterns of association rather than deriving exposure‐specific causal estimates, a single, theory‐driven confounder set was applied to all predictors, grounded in the ICF/Disablement Process Conceptual Model [11].

For the primary analysis, a fully adjusted model was fitted to the entire sample to estimate overall associations between predictors and disabling long COVID. This model aimed to describe the independent associations between each predictor and disabling long COVID, conditional on other covariates. These coefficients are not interpreted as causal effects but as adjusted associations, consistent with multivariable descriptive epidemiology. Model diagnostics were conducted to assess fit and stability. Multicollinearity was assessed using variance inflation factors (VIFs), which were acceptable (mean VIF = 3.42, all < 10). Goodness‐of‐fit testing using the Pearson chi‐square statistic indicated no overdispersion (*χ* ^2^(12,011) = 11,307.32; dispersion = 0.94; *p* > 0.99), supporting the use of Poisson regression with robust standard errors.

To further assess whether associations differed by sex, models were estimated separately for men and women. Sex‐based effect modification was then tested by including sex × covariate interaction terms in the full model and conducting joint Wald tests. The resulting *p*‐values were used to determine whether observed differences between men and women were statistically significant.

In a sensitivity analysis, the fully adjusted model was re‐estimated excluding self‐rated health and long‐standing illness, which may act as mediators. Analyses were conducted in Stata 18.0 with two‐sided tests and *p* < 0.05 as the significance threshold.

## 3. Results

Of the 12,033 participants included in the analytic sample, 690 (5.7%) reported disabling long COVID and 11,343 (94.3%) did not. Participants with disabling symptoms differed significantly from those without across multiple characteristics.

Descriptive characteristics varied significantly by disabling long COVID status (Table 1). Disabling symptoms were more common among adults aged 30–69 years, women and non‐White participants (all *p* < 0.01). Indicators of social and health disadvantage, including low income satisfaction, current smoking and pre‐existing health conditions, were more prevalent among those with disabling long COVID. Poor self‐rated health, high psychological distress and very bad sleep were also markedly more common in this group (all *p* < 0.001). In contrast, area of residence (urban vs. rural) did not differ significantly between groups (*p* = 0.739).

**TABLE 1:** Baseline characteristics of participants by disabling long COVID status.

Characteristics	Nondisabling LC (*n* = 11,343)	Disabling LC (*n* = 690)	All (*N* = 12,033)	*p*‐value
Age group, *n* (%)				< 0.001
16–29	2001 (17.6)	92 (13.3)	2093 (17.4)	
30–49	4152 (36.6)	290 (42.0)	4442 (36.9)	
50–69	4037 (35.6)	273 (39.6)	4310 (35.8)	
70+	1153 (10.2)	35 (5.1)	1188 (9.9)	
Sex, *n* (%)				< 0.001
Male	4730 (41.7)	227 (32.9)	4957 (41.2)	
Female	6613 (58.3)	463 (67.1)	7076 (58.8)	
Ethnicity, *n* (%)				0.005
White	9803 (86.4)	570 (82.6)	10,373 (86.2)	
Non‐White	1540 (13.6)	120 (17.4)	1660 (13.8)	
Residence, *n* (%)				0.739
Urban	8628 (76.1)	521 (75.5)	9149 (76.0)	
Rural	2715 (23.9)	169 (24.5)	2884 (24.0)	
Income satisfaction, *n* (%)				< 0.001
Low	2393 (21.1)	239 (34.6)	2632 (21.9)	
Moderate	3628 (32.0)	209 (30.3)	3837 (31.9)	
High	5000 (44.1)	225 (32.6)	5225 (43.4)	
Missing	322 (2.8)	17 (2.5)	339 (2.8)	
Current smoker, *n* (%)				0.010
Yes	1097 (9.7)	91 (13.2)	1188 (9.8)	
No	10,160 (89.6)	593 (85.9)	10,753 (89.4)	
Missing	86 (0.8)	6 (0.9)	92 (0.8)	
Pre‐existing health condition, *n* (%)				< 0.001
Yes	3467 (30.6)	323 (46.8)	3790 (31.5)	
No	7876 (69.4)	367 (53.2)	8243 (68.5)	
Self‐rated health, *n* (%)				< 0.001
Excellent/very good	5595 (49.3)	215 (31.2)	5810 (48.3)	
Good	3661 (32.3)	230 (33.3)	3891 (32.3)	
Fair/poor	1791 (15.8)	229 (33.2)	2020 (16.8)	
Missing	296 (2.6)	16 (2.3)	312 (2.6)	
Psychological distress, *n* (%)				< 0.001
GHQ‐12 caseness ≥ 4	1978 (17.4)	238 (34.4)	2216 (18.4)	
GHQ‐12 score < 4	8990 (79.3)	433 (62.8)	9423 (78.3)	
Missing	375 (3.3)	19 (2.8)	394 (3.3)	
Sleep quality, *n* (%)				< 0.001
Very good	2349 (20.7)	71 (10.3)	2420 (20.1)	
Fairly good	6467 (57.0)	344 (49.9)	6811 (56.6)	
Fairly bad	2038 (18.0)	210 (30.4)	2248 (18.7)	
Very bad	396 (3.5)	60 (8.7)	456 (3.8)	
Missing	93 (0.8)	5 (0.7)	98 (0.8)	

*Note:* Percentages are column percentages. *p*‐values are from chi‐square tests comparing participants with and without disabling long COVID. GHQ‐12 = 12‐item General Health Questionnaire.

Abbreviation: LC = long COVID.

**FIGURE 1:**
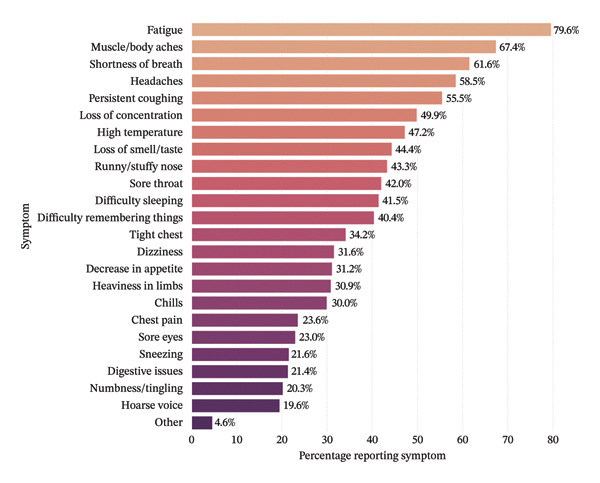
Frequency of reported symptoms among individuals reporting disabling long COVID.

Figure 1 illustrates the multisystem symptom profile of the 690 individuals with disabling long COVID. Fatigue was most common (79.6%), followed by muscle aches (67.4%), shortness of breath (61.6%), headaches (58.5%) and persistent cough (55.5%). Cognitive and systemic symptoms were also frequent: loss of concentration (49.9%), fever (47.2%) and loss of smell/taste (44.4%). Over 40% reported upper respiratory symptoms (runny nose and sore throat), sleep disturbance and memory problems. Other symptoms, such as dizziness, appetite loss and chills, clustered around 30%, while chest pain, digestive issues and numbness affected 19%–24%. Only 4.6% cited ‘other’ symptoms.

**FIGURE 2:**
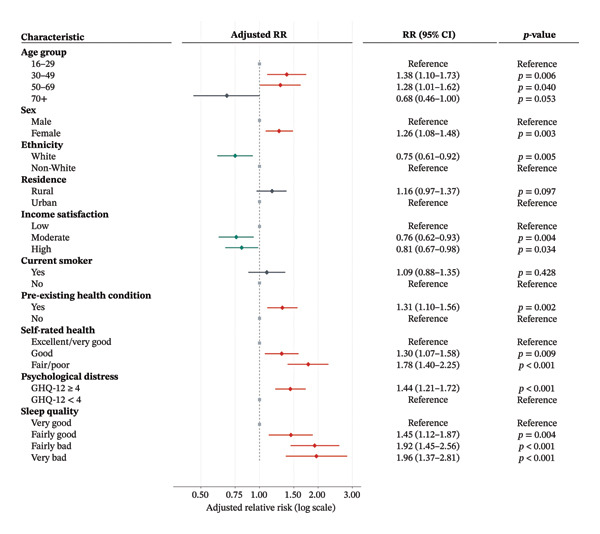
Factors associated with disabling long COVID: adjusted relative risks (RR) estimated from Poisson regression. *p* < 0.05 was considered statistically significant; adjusted models include mutual adjustment for all variables listed.

After adjusting for all covariates, many associations persisted, though attenuated. Middle‐aged adults remained at elevated risk (30–49: RR 1.38, 95% CI 1.10–1.73, *p* = 0.006; 50–69: RR 1.28, 95% CI 1.01–1.62, *p* = 0.040), while the protective effect in the oldest group weakened (≥ 70: RR 0.68, 95% CI 0.46–1.00, *p* = 0.053). Female sex was still a significant predictor (RR 1.26, 95% CI 1.08–1.48, *p* = 0.003), and White ethnicity conferred lower risk versus non‐White (RR 0.75, 95% CI 0.61–0.92, *p* = 0.005). Compared with low‐income satisfaction, moderate (RR 0.76, 95% CI 0.62–0.93, *p* = 0.004) and high satisfaction (RR 0.81, 95% CI 0.67–0.98, *p* = 0.034) were protective. A pre‐existing health condition increased adjusted risk by 31% (RR 1.31, 95% CI 1.10–1.56, *p* = 0.002), and fair/poor self‐rated health was associated with a nearly 80% higher risk (RR 1.78, 95% CI 1.40–2.25, *p* < 0.001). Psychological distress (GHQ‐12 ≥ 4) raised risk by 44% (RR 1.44, 95% CI 1.21–1.72, *p* < 0.001), and ‘fairly bad’ or ‘very bad’ baseline sleep each nearly doubled risk (RR 1.92, 95% CI 1.45–2.56 and RR 1.96, 95% CI 1.37–2.81; both *p* < 0.001). Neither current smoking (RR 1.09, 95% CI 0.88–1.35, *p* = 0.428) nor rural residence (RR 1.16, 95% CI 0.97–1.37, *p* = 0.097) remained significant (Figure 2).

### 3.1. Sex‐Stratified Analyses

Stratified analyses (Table 2) revealed differences in risk factors for disabling long COVID between men and women. Among men, middle age was associated with higher risk (30–49: RR 1.75, 95% CI 1.10–2.77, *p* = 0.018; 50–69: RR 1.72, 95% CI 1.08–2.75, *p* = 0.022), as were fair/poor self‐rated health (RR 1.57, 95% CI 1.04–2.37, *p* = 0.030), psychological distress (RR 1.44, 95% CI 1.00–2.08, *p* = 0.048) and poor sleep quality (fairly good: RR 1.78, 95% CI 1.15–2.77, *p* = 0.010; fairly bad: RR 2.13, 95% CI 1.26–3.59, *p* = 0.005; very bad: RR 2.42, 95% CI 1.18–4.94, *p* = 0.015).

**TABLE 2:** Factors associated with disabling long COVID stratified by sex.

Variable	RR (95% CI), men	*p*‐value, men	RR (95% CI), women	*p*‐value, women	*p* for interaction (sex)
Age group					0.095
16–29	Reference		Reference		
30–49	1.75 (1.10–2.77)	0.018	1.27 (0.98–1.66)	0.075	
50–69	1.72 (1.08–2.75)	0.022	1.16 (0.88–1.52)	0.290	
70+	1.28 (0.69–2.36)	0.428	0.45 (0.26–0.78)	0.004	
Ethnicity					0.184
Non‐White	Reference		Reference		
White	0.93 (0.62–1.37)	0.697	0.68 (0.54–0.86)	0.001	
Residence					0.011
Rural	0.82 (0.59–1.14)	0.243	1.35 (1.11–1.66)	0.003	
Urban	Reference		Reference		
Income satisfaction					0.009
Low	Reference		Reference		
Moderate	0.94 (0.67–1.32)	0.739	0.68 (0.54–0.85)	0.001	
High	0.72 (0.50–1.03)	0.073	0.86 (0.69–1.09)	0.217	
Current smoker					0.106
Yes	1.01 (0.68–1.50)	0.962	1.16 (0.90–1.51)	0.247	
No	Reference		Reference		
Pre‐existing health condition					0.757
Yes	1.27 (0.94–1.72)	0.127	1.34 (1.09–1.66)	0.006	
No	Reference		Reference		
Self‐rated health					0.061
Excellent/very good	Reference		Reference		
Good	1.02 (0.73–1.43)	0.900	1.46 (1.14–1.86)	0.002	
Fair/poor	1.57 (1.04–2.37)	0.030	1.87 (1.40–2.50)	< 0.001	
Psychological distress					0.972
GHQ‐12 caseness ≥ 4	1.44 (1.00–2.08)	0.048	1.44 (1.18–1.77)	< 0.001	
GHQ‐12 score < 4	Reference		Reference		
Sleep quality					0.651
Very good	Reference		Reference		
Fairly good	1.78 (1.15–2.77)	0.010	1.29 (0.94–1.77)	0.109	
Fairly bad	2.13 (1.26–3.59)	0.005	1.82 (1.29–2.55)	0.001	
Very bad	2.42 (1.18–4.94)	0.015	1.77 (1.17–2.67)	0.007	

*Note:* Relative risks (RR) and *p*‐values are shown separately for men and women. The final column reports the *p*‐value from Wald tests of sex × covariate interaction terms, indicating whether differences between men and women are statistically significant. Sex‐specific estimates may differ in magnitude, but only interactions with *p* < 0.05 provide evidence of effect modification by sex. *p* < 0.05 was considered statistically significant; adjusted models include mutual adjustment for all variables listed in the table.

In women, older age was protective (≥ 70: RR 0.45, 95% CI 0.26–0.78, *p* = 0.004), while White ethnicity conferred lower risk (RR 0.68, 95% CI 0.54–0.86, *p* = 0.001) and moderate income satisfaction was protective (RR 0.68, 95% CI 0.54–0.85, *p* = 0.001). Increased risk was observed for rural residence (RR 1.35, 95% CI 1.11–1.66, *p* = 0.003), pre‐existing health conditions (RR 1.34, 95% CI 1.09–1.66, *p* = 0.006), poorer self‐rated health (good: RR 1.46, 95% CI 1.14–1.86, *p* = 0.002; fair/poor: RR 1.87, 95% CI 1.40–2.50, *p* < 0.001), psychological distress (RR 1.44, 95% CI 1.18–1.77, *p* < 0.001) and poor sleep quality (fairly bad: RR 1.82, 95% CI 1.29–2.55, *p* = 0.001; very bad: RR 1.77, 95% CI 1.17–2.67, *p* = 0.007).

Formal interaction tests showed significant sex modification for residence (*p* for interaction = 0.011) and income satisfaction (*p* for interaction = 0.009), with adverse effects of rural living and protective effects of moderate income satisfaction limited to women. Weaker evidence suggested possible sex differences for age (*p* = 0.095) and self‐rated health (*p* = 0.061), while no interactions were observed for ethnicity, smoking, pre‐existing conditions, psychological distress, or sleep quality.

### 3.2. Sensitivity Analysis

Excluding self‐rated general health and long‐standing illness, both regarded as potential mediators, did not materially change the results. All key associations kept the same direction and statistical significance. Detailed estimates are provided in Supporting Table S1.

## 4. Discussion

This study identifies key prepandemic risk factors associated with the development of disabling long COVID, defined as persistent symptoms lasting more than twelve weeks that limit daily activities. Adults aged 30 to 69 years were more likely to experience disabling symptoms than those aged 16 to 29 years. Women were also more affected than men, consistent with previous observations of higher long COVID prevalence among females [14–16]. In addition to age and sex, several prepandemic factors were independently associated with greater risk, including lower satisfaction with income, non‐White ethnicity, pre‐existing long‐term health conditions, poor self‐rated health, psychological distress and poor sleep quality. These findings suggest that disabling long COVID does not affect all individuals equally and that socioeconomic and mental health vulnerabilities present before the pandemic may shape who experiences the most severe and functionally limiting outcomes.

The analysis revealed clear differences in risk profiles between men and women. Among men, middle age (30–69 years) was associated with significantly higher risk compared to younger men, whereas women in the same age groups showed elevated but nonsignificant estimates. In contrast, being aged 70 years or older was protective for women but not for men. Living in a rural area was associated with increased risk only among women, and moderate income satisfaction was protective in women but not in men, with both factors showing statistically significant sex interactions. Weaker evidence also suggested possible sex differences in the associations with age and self‐rated health. These sex‐specific patterns underline the need for tailored public health responses. Overall, the findings highlight that disabling long COVID is shaped not only by clinical factors but also by socioeconomic and structural determinants established before the pandemic.

A growing body of research has established that long COVID disproportionately affects certain demographic and clinical groups. Women, older adults and individuals with pre‐existing physical or mental health conditions have consistently been identified as being at higher risk of developing persistent symptoms following SARS‐CoV‐2 infection [17]. Several studies have also linked long COVID to socioeconomic disadvantage and elevated psychological stress [18]. However, most existing research has focused primarily on the presence or persistence of symptoms, rather than on their severity or impact on functional ability. This limits the ability to assess how long COVID interferes with individuals’ capacity to work, care for others or participate in everyday life—factors that are increasingly recognised as critical in public health and disability frameworks.

Furthermore, the majority of evidence to date comes from clinical cohorts, electronic health records or online surveys. These sources are valuable but often capture only those who seek care or have the means to participate in research, which may lead to selection bias and underrepresentation of marginalised populations. Few studies have used nationally representative data or been able to draw on comprehensive baseline information collected before the pandemic began [19]. As a result, it has been difficult to determine whether observed associations reflect true predisposing risk factors or are confounded by experiences during or after infection. This gap highlights the need for prospective, population‐based studies that can disentangle pre‐existing vulnerabilities from postinfection consequences and better inform prevention and support strategies.

This study makes a significant contribution by focusing specifically on disabling long COVID, defined as persistent symptoms that interfere with daily functioning, rather than on the broader and more heterogeneous category of long COVID symptoms alone. This distinction is important because not all individuals with lingering symptoms experience a loss of functional capacity [20, 21]. By operationalising disability in alignment with public health frameworks, such as those used by the UK ONS and the WHO’s ICF, this study offers a more meaningful measure for informing social policy, workplace accommodation and long‐term healthcare planning. It moves beyond simply counting symptoms and shifts attention towards the consequences of long COVID on people’s lives.

The symptom profile observed in this study is broadly consistent with existing literature, with fatigue, muscle aches and breathlessness predominating. However, it is worth noting that these findings differ somewhat from the 2024 RECOVER‐Adult Long COVID Research Index (LCRI), which assigns a relatively high diagnostic weight to changes in smell or taste [22]. In the LCRI, symptom weights were derived using LASSO regression to identify symptoms that best discriminate between SARS‐CoV‐2‐infected and uninfected participants, rather than reflecting raw symptom prevalence. Thus, the high weight assigned to smell/taste changes in the LCRI reflects their specificity to SARS‐CoV‐2 infection, not necessarily their frequency among those with long COVID. In this sample, loss of smell or taste was reported by 44.4% of individuals with disabling long COVID, ranking below fatigue, muscle aches, breathlessness, headaches and persistent cough. This difference likely also reflects the predominance of Omicron‐era infections in the follow‐up period (2022‐23), during which olfactory symptoms became less common compared with earlier variants [23]. Additionally, because the UKHLS only collected detailed symptom data from participants who reported long COVID lasting more than 12 weeks with activity limitation, it was not possible to compare symptom profiles between those with disabling long COVID, those with nondisabling long COVID and those without long COVID. Future research with symptom data across the full spectrum of postinfection outcomes would be valuable for identifying whether specific symptoms, such as loss of smell or taste, may distinguish disabling from nondisabling forms of long COVID.

Unlike many previous studies that rely on clinical or convenience samples, this analysis is based on a large cohort with rich, prepandemic baseline data. This design allows for a clearer identification of risk factors rather than consequences. While prior research has suggested associations between long COVID and mental health, socioeconomic status or health behaviours [16–21], few studies have been able to confirm whether these factors pre‐dated infection or emerged afterwards. By prospectively linking data from before the pandemic with outcomes measured three to 4 years later, this study provides stronger evidence that psychological distress, sleep problems and financial dissatisfaction were already present and independently predicted disabling long COVID. This is a key methodological advancement, enabling the identification of individuals who may be more vulnerable to prolonged and disabling recovery trajectories.

Importantly, this study also offers new insights into sex‐specific risk patterns. While women overall had a higher risk of disabling long COVID, stratified analyses revealed that the role of age, rural or urban residence and health perceptions varied by sex. Older age appeared to be protective only among women, while rural residence increased risk only for women, suggesting that social roles, caregiving responsibilities or healthcare access may interact with sex to shape long COVID outcomes. These nuanced findings contrast with many earlier studies that report pooled estimates, potentially obscuring meaningful subgroup differences [17–19]. By highlighting these interactions, this study underlines the need for tailored interventions and policies that consider the intersection of sex, health and social determinants in shaping the long‐term impact of the pandemic.

### 4.1. Limitations of This Study

Several limitations should be noted. First, disabling long COVID was based on self‐report and may be influenced by recall or reporting bias. Second, the analysis did not include information on the severity of initial infection or vaccination status, which may influence risk. Third, although the sample is broadly representative of the UK population, certain high‐risk groups (e.g. those with severe disability or limited digital access) may still be underrepresented. Fourth, symptom data were collected only for participants who reported disabling long COVID, which precluded comparison of symptom profiles across those with disabling, nondisabling or no long COVID; such comparisons could help identify symptoms that distinguish functional severity. Finally, the binary measure of functional limitation does not capture the full spectrum or duration of disability, which may fluctuate over time.

## 5. Conclusion

This study identifies several prepandemic factors associated with increased risk of developing disabling long COVID, including being aged 30 to 69 years, female sex, non‐White ethnicity, low satisfaction with income, pre‐existing health conditions, poorer self‐rated health, psychological distress and poor sleep quality. By using large prospective data, it offers stronger evidence that these vulnerabilities existed before infection and may contribute to prolonged functional impairment. The findings highlight the importance of distinguishing between symptom presence and disability in understanding the true burden of long COVID. They also reveal sex‐specific differences in risk, pointing to the need for more targeted and equitable public health responses. Overall, addressing the social and health inequalities that shape susceptibility to disabling long COVID should be a central priority in ongoing recovery planning.

## Funding

Yusuff Adebayo Adebisi was supported by a UKRI Economic and Social Research Council (ESRC) (ES/P000681/1).

## Ethics Statement

UKHLS has received ethics clearance for all waves from the University of Essex Ethics Committee and, for nurse/biomarker modules, the National Health Service Research Ethics Committee. This analysis uses fully anonymised, publicly available data (UK Data Service, SN 2000053); no additional institutional review board approval was required.

## Conflicts of Interest

The author declares no conflicts of interest.

## Supporting Information

Additional supporting information is available in the Supporting File, including Supporting Table S1, which presents the sensitivity analysis comparing the mutually adjusted model with a mediator‐stripped model.

## Supporting information


**Supporting Information** Additional supporting information can be found online in the Supporting Information section.

## Data Availability

The data analysed in this study are publicly available from the University of Essex, Institute for Social and Economic Research, via the UK Data Service. Understanding Society (data series), 13th release, 2024. SN: 2000053. https://doi.org/10.5255/UKDA-Series-2000053.
