# Supplementary material for: Prepandemic Risk Factors for Disabling Long COVID: A Prospective Cohort Analysis
Source: J Trop Med. 2026 Apr 10;2026:9396282. doi: 10.1155/jotm/9396282 (PMC13069177; doi:10.1155/jotm/9396282)
Supplement: Supplementary file 1 — Supporting Information Additional supporting information can be found online in the Supporting Information section. [file JOTM-2026-9396282-s001.pdf]

Pre-Pandemic Risk Factors for Disabling Long COVID: A Prospective Cohort Analysis

Yusuff Adebayo Adebisi[1,2]

1. College of Social Sciences, University of Glasgow, Glasgow, UK
2. Scottish Centre for Administrative Data Research, University of Glasgow, UK

Corresponding Author: Yusuff Adebayo Adebisi; College of Social Sciences, University of Glasgow, 40 Bute Gardens, Glasgow, G12 8RT, United Kingdom. Email: y.adebisi.1@research.gla.ac.uk

Supplementary Table S1. Sensitivity analysis comparing the mutually adjusted model with a mediator-stripped model: incidence-rate ratios (IRRs) for disabling Long COVID

Predictor (reference category)	Main model† RR (95 % CI)	Sensitivity model‡ RR (95 % CI)
**Age group** (ref 16–29)		
30–49	1.38 (1.10 – 1.73)	1.44 (1.15 – 1.82)
50–69	1.28 (1.01 – 1.62)	1.49 (1.18 – 1.89)
70 +	0.68 (0.46 – 1.00)	0.88 (0.60 – 1.29)
**Sex** (ref male)		
Female	1.26 (1.08 – 1.48)	1.26 (1.08 – 1.48)
**Ethnicity** (ref Non-white)		
White	0.75 (0.61 – 0.92)	0.75 (0.61 – 0.91)
**Residence** (ref urban)		
Rural	1.16 (0.97 – 1.37)	1.15 (0.97 – 1.37)
**Income satisfaction** (ref low)		
Moderate	0.76 (0.62 – 0.93)	0.73 (0.61 – 0.88)
High	0.81 (0.67 – 0.98)	0.70 (0.57 – 0.85)
**Smoking status** (ref no)		
Current smoker	1.09 (0.88 – 1.35)	1.19 (0.96 – 1.48)
**Psychological distress** (ref score < 4)		
GHQ-12 ≥ 4	1.44 (1.21 – 1.72)	1.64 (1.38 – 1.95)
**Sleep quality** (ref very good)		
Fairly good	1.45 (1.12 – 1.87)	1.57 (1.22 – 2.02)
Fairly bad	1.92 (1.45 – 2.56)	2.31 (1.75 – 3.05)
Very bad	1.96 (1.37 – 2.81)	2.68 (1.89 – 3.79)

† Main model mutually adjusted for age group, sex, ethnicity, residence, income satisfaction, smoking, pre-existing health conditions, self-rated health, psychological distress, and sleep quality.
‡ Sensitivity model excludes self-rated health and pre-existing health conditions.
